# The Interaction between Sex and Hyperlipidemia on Gout Risk Is Modulated by HLA-B Polymorphic Variants in Adult Taiwanese

**DOI:** 10.3390/genes10030246

**Published:** 2019-03-25

**Authors:** Tsui-Wen Hsu, Pei-Shyuan Lee, Oswald Ndi Nfor, Chia-long Lee, Pei-Hsin Chen, Disline Manli Tantoh, Long-Yau Lin, Ming-Chih Chou, Yu-Chen Lee, Yung-Po Liaw

**Affiliations:** 1 Institute of Medicine, Chung Shan Medical University, Taichung 40201, Taiwan; irb@cgh.org.tw; 2 Superintendent Office, Cathay General Hospital, Taipei 106, Taiwan; 3 Department of Family Medicine, Neihu Clinic, Attending physician, Cathay General Hospital, Taipei 106, Taiwan; cghleeps@gmail.com; 4 Department of Public Health and Institute of Public Health, Chung Shan Medical University, Taichung 40201, Taiwan; nforoswald2@yahoo.com (O.N.N.); c0701chen@gmail.com (P.-H.C.); tantohdisline@yahoo.com (D.M.T.); 5 Department of Internal Medicine, Cathy General Hospital, Taipei 106, Taiwan; cghleecl@hotmail.com; 6 School of Medicine, Chung Shan Medical University, Taichung 40201, Taiwan; xillin681113@gmail.com; 7 Department of Dentistry, Cathy General Hospital, Taipei 106, Taiwan; 8 Department of Family and Community Medicine, Chung Shan Medical University Hospital, Taichung 40201, Taiwan

**Keywords:** gout, hyperlipidemia, sex

## Abstract

The effects of genetic variants on the interaction between hyperlipidemia and sex have not been investigated among gout patients in Taiwan. Using Taiwan Biobank and the National Health Insurance Research Database (NHIRD), we examined hyperlipidemia, sex, and their relationship with gout among Taiwanese adults with the human leukocyte antigen B (HLA-B) genetic variants. Hyperlipidemia was present in 1437 patients with gout. Sex and hyperlipidemia had significant associations on gout risk, with hyperlipidemia showing a relatively stronger effect. Gout was present in men, with an odds ratio (OR) of 1.945 (95% confidence interval (CI) 1.568–2.411) compared to women, and in hyperlipidemic (OR = 4.032; 95% CI: 3.581–4.540) compared to non-hyperlipidemic patients. The interaction of sex and hyperlipidemia was significant for rs2523608 GG (*p* = 0.0402) and rs4713518 AA (*p* = 0.0003) genotypes. After stratification, hyperlipidemia remained a risk factor in women (OR = 4.735, 95% CI: 3.375–6.643) and men (OR = 3.640, 95% CI: 2.916–4.544) with rs2523608 GG genotype. The odds ratio in hyperlipidemic women and men with rs4713518 AA genotype was 7.454 (95% CI 5.103–10.888) and 3.585 (95% CI 2.854–4.503), respectively. Our study indicates that hyperlipidemia-sex interactions exist for gout risk in Taiwanese adults with rs2523608 GG and rs4713518 AA genotypes.

## 1. Introduction

Gout is the most prevalent form of inflammatory arthropathy and is often associated with hyperuricemia [1,2]. It is caused by a chronic elevation of serum uric acid levels above the saturation point for monosodium urate crystal (MSU) formation. The deposition of MSU crystals, which occurs predominantly in peripheral joints and surrounding tissues, defines gout [3]. Gout is common in most countries in North America and Western Europe, with prevalence ranging from 1–4% [4]. However, its incidence and prevalence have a distinct geographical and racial distribution [4]. Taiwan is one of the countries with the highest prevalence of gout [5]. The prevalence was 6.24% and incidence was 2.74 per 1000 person-years in 2010 [6]. According to the 2005–2008 Nutrition and Health Survey in Taiwan (NAHSIT), the prevalence of gout is higher in older adults and men in particular [5]. 

Several risk factors such as age, sex, obesity, body mass index, dietary factors, diabetes, and hypertension contribute to the development of gout [7]. There is a high prevalence of obesity, hyperlipidemia, and hypertension in Taiwan. Hyperlipidemia, a well-established risk factor for CVD has also been associated with gout. According to the NAHSIT 2014–2017 survey, the prevalence of hyperlipidemia was 21.76% in men (23.80%) and 19.78% in women. About 77% of patients with primary gout were observed with hyperlipidemia [8].

Previous findings suggested that women with gout might suffer from more gout-related comorbidities than men [9]. Hyperlipidemia is one of the significant cardiovascular comorbidities of rheumatoid arthritis in Taiwan [10]. Significant associations have been found between hyperlipidemia and hyperuricemia [11], both of which are suggested to be more common in men compared to women. 

Gout treatment and prevention involves the use of allopurinol. However, this drug has been associated with severe cutaneous adverse reactions (SCAR) in Han Chinese [12]. Furthermore, allopurinol-SCAR showed a significant association with the human leukocyte antigen (HLA) alleles. In light of this, testing for HLA polymorphic variants has been recommended for gout patients. 

Previous studies investigating risk factors for gout in Taiwan have focused mainly on hyperuricemia. There is no previously published work on genetic predisposition, hyperlipidemia, and gout. Therefore, the aim of this study was to investigate the associations of gout with hyperlipidemia and sex among carriers of HLA-B gene variants (rs2523608 and rs4713518) in Taiwan.

## 2. Materials and Methods 

### 2.1. Data Source

Study data were obtained from two data sources: (1) The National Health Insurance Research Database and (2) Taiwan Biobank, a national health resource that contains genetic information of over 200,000 Taiwanese residents aged 30 to 70 years. Presently, there are 29 recruitment centers with each city or county having at least one. Recruitment methods in the Taiwan Biobank are in accordance with relevant guidelines and regulations. Written informed consents are obtained from all participants prior to data collection. Both data sources were linked and information about gout and hyperlipidemia was obtained using personal identification numbers. The study was conducted in accordance with the Declaration of Helsinki, and the protocol was approved by the Institutional Review Board of Chung Shan Medical University.

### 2.2. Study Participants

Overall, 16,878 individuals consisting of 7974 men and 8904 women aged 30–70 years were included in the current study. Information on their demographic (sex, age, body mass index [BMI], waist-hip ratio [WHR], and body fat), biochemical (creatinine and uric acid), lifestyle (regularly exercise, smoking and alcohol consumption), and disease history (hyperlipidemia, diabetes and hypertension) was retrieved from the biobank database. Categorization of participants was based on sex and hyperlipidemia. Participants were defined as having gout or hyperlipidemia if they had either two-outpatient visits or one-time hospitalization with reported International Classification of Diseases, Ninth Revision, Clinical Modification (ICD-9 CM) 274 and 272, respectively.

#### Genetic Variant Selection

A search of peered-reviewed literature databases was carried out to select the HLA polymorphic variants (rs2523608 and rs4713518) associated with gout. The search terms included gout, Taiwan, HLA variants, and allopurinol. Genotyping was performed at the National Center for Genome Medicine in Academia Sinica using the Axiom-Taiwan Biobank Array Plate (Affymetrix, Santa Clara, CA, USA). We included only participants with call rates greater than 90% were. Polymorphic variants with minor allele frequency (MAF) <0.05, as well as those whose genotypes deviated from the Hardy-Weinberg equilibrium (HWE) were excluded.

### 2.3. Statistical Analysis 

Data were managed and analyzed with the SAS 9.4 software (SAS Institute, Cary, NC, USA). Differences between gout statuses were evaluated using a t-test for continuous variables and Chi-square test for categorical variables. Multivariate logistic regression models were used to weigh the association of Gout with hyperlipidemia and the investigated variants. Data were presented as mean ± standard error for continuous variables. Odds ratio (OR) and 95% confidence intervals (CI) were estimated.

## 3. Results

Table 1 shows the baseline characteristics of study participants. The study comprised of 2182 individuals with gout and 14,696 control individuals. Among those with gout, 586 (26.86%) were women while 1596 (73.14%) were men. Participants with gout were older (mean ± SE, 53.113 ± 0.219 years vs. 47.970 ± 0.090 years, *p* < 0.0001) and had a higher concentration of uric acid (6.832 ± 0.037 mg/dl vs. 5.447 ± 0.011 mg/dl. Participants with and without gout were significantly different (*p* < 0.0001). Hyperlipidemia was present in 1437 patients with gout. After adjusting for potential confounders, sex and hyperlipidemia both had significant effects on gout risk (Table 2). Hyperlipidemia appeared to show a greater effect than sex. Gout was present in men, with an odds ratio (OR) of 1.945 (95% confidence interval (CI) 1.568–2.411) compared to women, and in hyperlipidemic (OR = 4.032; 95% CI: 3.581–4.540) compared to non-hyperlipidemic patients. Uric acid was associated with a higher risk of gout (OR = 1.677; 95% CI: 1.609–1.748). Significant odds ratios were also found for diabetes (OR = 1.555, 95% CI: 1.363–1.772) and hypertension (OR = 1.249, 95% CI: 1.107–1.409). The addition of HLA-B polymorphic variants into the model did not affect gout risk in men and hyperlipidemic patients (Table 3). However, when the dominant model was applied, the interaction of sex and hyperlipidemia on gout risk was significant for rs2523608 GG genotype (P_interaction_ = 0.0402) and rs4713518 AA genotype (P_interaction_ < 0.0003). After stratification based on rs2523608 GG and rs4713518 AA genotypes (Table 4), hyperlipidemia remained a risk factor for gout in women (OR = 4.735, 95% CI: 3.375–6.643) and men (OR = 3.640, 95% CI: 2.916–4.544) with rs2523608 GG genotype. The odds ratio for gout in hyperlipidemic women and men with rs4713518 AA genotype was 7.454 (95% CI: 5.103–10.888) and 3.585 (95% CI: 2.854–4.503), respectively.

## 4. Discussion

We have provided knowledge related to sex and hyperlipidemia in gout risk among Taiwanese adults aged 30–70 years. To date, such an investigation has not been reported in Taiwan. Using data obtained from Taiwan Biobank, we found that: (1) Consistent with previous findings, gout risk was higher in Taiwanese men than women [13]; (2) hyperlipidemia was associated with gout risk; and (3) there was a significant interaction between hyperlipidemia and sex among carriers of rs2523608 GG and rs4713518 AA genotypes. Hyperuricemia, an important risk factor for gout development is more common in men than women [14]. However, patients with gouts do not necessarily have hyperuricemia [15]. Because hyperlipidemia, particularly hypertriglyceridemia, has been associated with hyperuricemia [11], we were prompted to analyze hyperlipidemia as an independent factor to determine its level of association or interaction with gout. As shown above, we found a significant association with gout. The mechanism of this association is not fully understood. However, this may be linked to abnormal regulation of carbohydrate metabolism in gouty patients [11]. 

Ethnic differences exist in the prevalence and incidence of gout. Previous findings indicated a higher prevalence of gout in black compared to white (5.0% vs. 4.0%) Americans [13]. In Taiwan, the prevalence was 6.24% in 2010 [6]. Sex and ethnic differences in gout risk have been attributed to co-morbidities [16]. In this study, we found that gout risk remained higher in Taiwanese men compared to women, after HLA-B genetic variants were included in the model. In the model, we found that the interaction between sex and hyperlipidemia was significant only for rs2523608 GG (P_interaction_ = 0.0402) and rs4713518 AA genotype (P_interaction_ = 0.0003). Further analysis was made based on these genotypes to better understand their effect on hyperlipidemia and gout risk in men and women. Results showed that hyperlipidemic women with both genotypes (that is, and rs2523608 GG and rs4713518 AA) had relatively higher odds ratios than men. That is (OR = 4.735, 95% CI 3.375–6.643 vs. 3.640, 95% CI 2.916–4.544 for rs2523608 GG and 7.454, 95% CI 5.103–10.888 vs. 3.585, 95% CI 2.854–4.503, respectively). Further investigation is necessary to clarify the possible mechanisms through which the interaction between sex and hyperlipidemia on gout risk is modulated by these single nucleotide polymorphisms (SNPs) in HLA-B gene.

Previous findings have shown that hyperlipidemia and hyperuricemia are more prevalent in men than women [17,18]. Other studies have also indicated that a gout patient profile is different in women compared to men [19,20]. After stratification by sex and HLA-B variants of interest, we found that hyperlipidemia remained a significant risk factor for gout, with a higher odds ratio found in women as stated above. Moreover, we found that uric acid was associated with a relatively higher odds ratio in men no matter the genotype. In addition, obesity was associated with a higher odds ratio (though not significant) in women with both genotypes, and in men with rs4713518. The association of gout with hypertension was significant only in men with both genotypes.

To date, no hyperlipidemia–sex findings have been reported in Taiwan. Interestingly in our findings, we found that there was an interaction between hyperlipidemia and sex on gout risk among specific HLA-B genotypes, even though the mechanism of such an association remains unclear. Therefore, larger studies would help to clarify these interactions. Our study is limited in that the study was restricted to participants aged 30–70 years. Therefore, younger and older people with gout were not included in the model. Nevertheless, we used a relatively large sample size.

In conclusion, we found that there was an interaction between hyperlipidemia and sex on gout risk among Taiwanese adults with rs2523608 GG and rs4713518 AA genotypes. Hyperlipidemia and sex were both associated with gout risk, with hyperlipidemia showing a greater impact. 

## Figures and Tables

**Table 1:** Baseline characteristics of study participants.

	No Gout	Gout	*p*-Value
	(n = 14696)	(n = 2182)	
Sex, n (%)			<0.0001
Female	8318(56.60%)	586(26.86%)	
Male	6378(43.40%)	1596(73.14%)	
Hyperlipidemia, n (%)			<0.0001
No	11149(75.86%)	745(34.14%)	
Yes	3547(24.14%)	1437(65.86%)	
Age, years (SE)	47.970 ± 0.090	53.113 ± 0.219	<0.0001
Exercise n (%)			<0.0001
No	8837(60.13%)	1130(51.79%)	
Yes	5859(39.87%)	1052(48.21%)	
BMI, n (%)			<0.0001
Normal	7551(51.83%)	667(30.57%)	
Underweight	501(3.41%)	19(0.87%)	
Overweight	4063(27.65%)	755(34.60%)	
Obesity, n (%)	2581(17.56%)	741(33.96%)	
Body fat rate, (%)	27.485 ± 0.061	27.082 ±0.162	0.0202
Smoking, n (%)			<0.0001
Never	11550(78.59%)	1443(66.13%)	
Former	1559(10.61%)	438(20.07%)	
Current	1587(10.80%)	301(13.79%)	
Drinking, n (%)			<0.0001
Never	13683(93.11%)	1910(87.53%)	
Current	1013(6.89%)	272(12.47%)	
Creatinine, mg/dL (SE)	0.727 ± 0.002	0.885 ± 0.011	<0.0001
Uric acid, mg/dL (SE)	5.447 ± 0.011	6.832 ± 0.037	<0.0001
Diabetes n, (%)			<0.0001
No	13122(89.29%)	1539(70.53%)	
Yes	1574(10.71%)	643(29.47%)	
Hypertension, n (%)			<0.0001
No	11925(81.14%)	1162(53.25%)	
Yes	2771(18.86%)	1020(46.75%)	

BMI, body mass index, SE, standard error.

**Table 2:** Association of sex and hyperlipidemia with gout.

	OR	95% C.I.
Sex (ref: Female)		
Male	1.945	1.568–2.411
Hyperlipidemia (ref: No)		
Yes	4.032	3.581–4.540
Age	1.002	1.016–1.028
Exercise (ref: No)		
Yes	0.974	0.873–1.087
BMI (ref: Normal)		
Underweight	0.944	0.569–1.567
Overweight	1.027	0.890–1.185
Obesity	1.282	1.056–1.557
Body fat rate	1.008	0.994–1.023
Smoking (ref: Never)		
Former	0.974	0.841–1.128
Current	0.835	0.708–0.984
Drinking (ref: Never)		
Current	1.088	0.917–1.291
Creatinine	1.201	1.017–1.417
Uric acid	1.677	1.609–1.748
Diabetes (ref: No)		
Yes	1.555	1.363–1.772
Hypertension (ref: No)		
Yes	1.249	1.107–1.409
Sex × hyperlipidemia	P_interaction_ = 0.0135	
OR = odds ratio, CI = 95% confidence interval, BMI = body mass index		

**Table 3:** Association of sex and hyperlipidemia with gout risk among carriers of HLA-B polymorphic variants.

	rs2523608				rs4713518			
	Normal (GG)		Mutation (AG+AA)		Normal (AA)		Mutation (GA+GG)	
	OR	95%C.I.	OR	95%C.I.	OR	95%C.I.	OR	95%C.I.
Sex (ref: Women)								
Men	1.872	1.348–2.600	1.958	1.469–2.610	1.374	0.969–1.946	2.411	1.833–3.172
Hyperlipidemia (ref: No)								
Yes	3.932	3.275–4.721	4.120	3.523–4.818	4.259	3.523–5.150	3.897	3.345–4.540
Age	1.024	1.015–1.033	1.020	1.012–1.028	1.016	1.007–1.026	1.025	1.017–1.033
Exercise (ref: No)								
Yes	0.857	0.723–1.017	1.069	0.925–1.234	1.068	0.898–1.270	0.916	0.795–1.056
BMI (ref: Normal)								
Underweight	0.949	0.433–2.078	0.935	0.481–1.818	0.758	0.344–1.670	1.092	0.562–2.118
Overweight	0.968	0.779–1.204	1.080	0.893–1.306	1.206	0.960–1.515	0.932	0.775–1.120
Obesity	1.015	0.755–1.365	1.521	1.175–1.969	1.432	1.048–1.955	1.204	0.939–1.544
Body fat rate	1.008	0.997–1.040	1.001	0.982–1.020	0.985	0.962–1.007	1.023	1.005–1.041
Smoking (ref: Never)								
Former	1.040	0.826–1.310	0.929	0.767–1.124	1.145	0.910–1.441	0.874	0.721–1.058
Current	0.791	0.614–1.018	0.865	0.696–1.075	0.904	0.687–1.189	0.798	0.649–0.981
Drinking (ref: Never)								
Current	1.203	0.922–1.570	1.019	0.815–1.275	1.097	0.837–1.438	1.078	0.864–1.345
Creatinine	1.143	0.937–1.394	1.320	0.972–1.794	1.065	0.801–1.417	1.279	1.020–1.604
Uric acid	1.639	1.539–1.746	1.705	1.613–1.802	1.725	1.612–1.846	1.656	1.572–1.745
Diabetes (ref: No)								
Yes	1.375	1.125–1.682	1.715	1.441–2.040	1.664	1.351–2.048	1.502	1.267–1.780
Hypertension (ref: No)								
Yes	1.316	1.093–1.584	1.189	1.013–1.395	1.382	1.142–1.673	1.163	0.994–1.360
Sex × hyperlipidemia	P_interaction_ = 0.0402		P_interaction_ = 0.1315		P_interaction_ = 0.0003		P_interaction_ = 0.8222	

**Table 4:** Association of gout with hyperlipidemia in Taiwanese men and women based on rs2523608 GG and rs4713518 AA genotypes.

	rs2523608 (Normal, GG)				rs4713518 (Normal, AA)			
	Women		Men		Women		Men	
	OR	95%C.I.	OR	95%C.I.	OR	95%C.I.	OR	95%C.I.
Hyperlipidemia (ref: No)								
Yes	4.735	3.375–6.643	3.640	2.916–4.544	7.454	5.103–10.888	3.585	2.854–4.503
Age	1.041	1.024–1.059	1.017	1.006–1.028	1.022	1.005–1.040	1.014	1.003–1.026
Exercise (ref: No)								
Yes	0.766	0.573–1.002	0.911	0.738–1.125	0.860	0.635–1.165	1.160	0.939–1.433
BMI (ref: Normal)								
Underweight	0.687	0.200–2.362	1.224	0.421–3.564	0.862	0.283–2.629	0.696	0.226–2.140
Overweight	1.068	0.728–1.621	0.903	0.695–1.173	1.233	0.804–1.890	1.200	0.914–1.576
Obesity	1.291	0.731–2.282	0.892	0.629–1.263	1.514	0.830–2.762	1.397	0.966–2.020
Body fat rate	1.025	0.986–1.064	1.014	0.987–1.041	0.995	0.955–1.036	0.978	0.950–1.007
Smoking (ref: Never)								
Former	0.562	0.161–1.958	1.111	0.875–1.410	0.367	0.082–1.648	1.235	0.972–1.568
Current	0.701	0.253–1.941	0.809	0.621–1.053	0.597	0.194–1.832	0.943	0.709–1.256
Drinking (ref: Never)								
Current	1.891	0.721–4.955	1.154	0.875–1.523	1.807	0.702–4.654	1.043	0.786–1.385
Creatinine	0.981	0.617–1.558	1.199	0.936–1.537	0.750	0.366–1.540	1.250	0.834–1.874
Uric acid	1.365	1.208–1.543	1.737	1.609–1.876	1.349	1.189–1.531	1.891	1.736–2.060
Diabetes (ref: No)								
Yes	1.591	1.160–2.182	1.272	0.980–1.652	1.674	1.213–2.311	1.613	1.230–2.115
Hypertension (ref: No)								
Yes	1.027	0.745–1.416	1.533	1.220–1.926	1.283	0.926–1.777	1.497	1.18–1.892
